# Medication-Related Osteonecrosis of the Jaw: A Cross-Sectional Survey among Urologists in Switzerland, Germany, and Austria

**DOI:** 10.3390/jcm12020638

**Published:** 2023-01-13

**Authors:** Salvatore Calderaro, Kathrin Bausch, Céline Tourbier, Christian Wetterauer, Florian M. Thieringer, Britt-Isabelle Berg

**Affiliations:** 1 Department of Oral and Cranio-Maxillofacial Surgery, University Hospital Basel, 4031 Basel, Switzerland; 2 Faculty of Medicine, University of Basel, 4031 Basel, Switzerland; 3 Department of Urology, University Hospital Basel, 4031 Basel, Switzerland; 4 Medical Additive Manufacturing Research Group (Swiss MAM), Department of Biomedical Engineering, University of Basel, 4123 Allschwil, Switzerland; 5 Department of Medicine, Faculty of Medicine and Dentistry, Danube Private University, 3500 Krems, Austria

**Keywords:** medication-related osteonecrosis of the jaw, MRONJ, bisphosphonates, antiresorptive agents, angiogenic inhibitors, knowledge, questionnaire, survey, urology

## Abstract

Medication-related osteonecrosis of the jaw (MRONJ) is a potentially preventable adverse side effect of mainly antiresorptive drugs. MRONJ is expected to become a growing clinical problem due to the aging population and the increasing number of patients requiring antiresorptive agents. Knowledge and awareness about MRONJ and elimination of the oral and dental risk factors before starting antiresorptive therapy (AR) are fundamental to reducing the incidence of MRONJ. In urology, ARs are used primarily in patients suffering from bone metastases due to prostate cancer and to prevent cancer-treatment-induced bone loss (CTIBL) in prostate cancer patients receiving endocrine therapy. This postal survey aimed to evaluate disease-related knowledge and awareness about implementing oral examinations for patients starting AR among Swiss, German, and Austrian urologists. A total of 176 urologists returned the completed questionnaire, yielding a response rate of 11.7%. Of the respondents, 44.9% (*n* = 79) and 24.4% (*n* = 43) stated that they give more than five first-time prescriptions of denosumab and of intravenous or oral bisphosphonates per year, respectively. Only 14.8% (*n* = 26) of the participating urologists had never encountered MRONJ cases related to BPs. Of the participants, 89.8% (*n* = 158) had implemented referrals to dentists for oral examination before initiating AR. The mean percentage of correct answers regarding the knowledge about MRONJ was 70.9% ± 11.2%. In contrast to previous surveys on MRONJ among physicians, this study showed that the participating urologists were sufficiently informed about MRONJ, as reflected by the high number of participants implementing preventive dental screenings.

## 1. Introduction

Medication-related osteonecrosis of the jaw (MRONJ) is a potentially severe condition that can dramatically impair masticatory function, speech, and swallowing, adversely affecting a patient’s oral-health-related quality of life. MRONJ is characterized by nonhealing necrotic jaw bone in patients with current or previous antiresorptive or antiangiogenic treatment without previous radiation therapy in the head or neck region [1]. The most common clinical manifestation of MRONJ is an exposed jaw bone; however, cases of MRONJ with nonexposed bone have been described [2,3,4]. Other symptoms may include dull bone pain in the jaw, loosening of teeth, signs of infection and inflammation, altered neurosensory function, and halitosis [5]. The incidence of MRONJ ranges from 0 to 12,222 per 100,000 patient-years, depending on the type of drug, dosage, and treatment purpose [6].

Antiresorptive and antiangiogenic agents are among the most prescribed drugs for the treatment or prevention of resorptive bone diseases such as inter alia multiple myeloma, bone metastases in the context of prostate, breast, and lung cancers, primary and secondary osteoporosis, Paget’s disease, and osteogenesis imperfecta [7,8,9,10,11,12].

Bisphosphonates (BPs) strongly inhibit osteoclast activity by inducing osteoclast apoptosis via the melanovate pathway. Consequently, bone formation exceeds bone resorption, improving bone density and significantly reducing the risk of skeletal-related events (SREs) [13,14]. Denosumab, another antiresorptive agent, is a human monoclonal antireceptor activator of nuclear factor-κB ligand (RANKL) antibody that inhibits the survival and differentiation of osteoclasts by blocking the RANKL/RANK interaction [15]. In addition to antiresorptive agents, antiangiogenic drugs such as vascular endothelial growth factor (VEGF) inhibitors (e.g., bevacizumab) and tyrosine kinase inhibitors (e.g., sunitinib) have been reported to induce MRONJ [16,17,18]. Recently, other agents such as mechanistic target of rapamycin (mTOR) inhibitors (e.g., everolimus), selective estrogen receptor modulators (SERMs) (e.g., raloxifene), fusion proteins (e.g., aflibercept), and antitumor necrosis factor (TNF) antibodies have been reported to induce MRONJ possibly. However, robust data are lacking [19].

Although some reports show that MRONJ can develop spontaneously without a clear initiating cause, many risk factors are associated with the development of MRONJ, such as intravenous (IV) administration of BPs, frequency of administration, higher dose per administration, and the duration of drug intake [20,21]. In addition, the use of corticosteroids and concomitant systemic diseases, such as diabetes mellitus, hypertension, renal failure, and immunosuppression, enhance the likelihood of developing MRONJ [22,23,24]. Furthermore, invasive dental measures (e.g., tooth extraction), preexisting periodontal inflammation, and prosthesis-related oral mucosal lesions represent significant risk factors for MRONJ, as has also been demonstrated in preclinical research using primarily rodent models [25,26,27,28].

The management of patients with MRONJ remains challenging, as this disease often requires long-term treatment [29]. Additionally, due to the increasing aging population and the growing number of cancer patients requiring antiresorptive and antiangiogenic medication, the number of MRONJ patients is expected to increase accordingly. This emphasizes the importance of the prevention and early diagnosis of MRONJ, which can only be achieved by a multidisciplinary approach involving physicians, dentists, and oral and maxillofacial surgeons or dental surgeons [30,31]. Moreover, the effectiveness of prevention strongly depends on knowledge about MRONJ and its risk factors among physicians and dentists.

Bone is a frequent site of metastasis in patients with prostate cancer. The treatment is often accompanied by the administration of high-dose BPs or denosumab to prevent SREs and low-dose BPs or denosumab to prevent other complications, such as cancer-therapy-induced bone loss (CTIBL) [32]. Given the rising incidence of metastatic prostate cancer patients requiring BPs and antiangiogenic agents [33,34], we aimed to evaluate the knowledge about MRONJ and the awareness about implementing preventive dental screenings among Swiss, German, and Austrian urologists.

## 2. Materials and Methods

For this cross-sectional study, a postal survey was conducted among 1500 urologists practicing in the German-speaking part of Switzerland, Germany, and Austria. The questionnaires were sent out on 3 May 2022, and the responses were collected until 17 June 2022. The publicly available postal addresses were retrieved from membership rosters on the websites of the Swiss Society of Urology, the German Society of Urology, and the Austrian Medical Association. Postal addresses of 369 Swiss and 518 Austrian urologists were found. Due to feasibility considerations, a random sample of 613 German urologists was extracted to survey a total number of 1500 urologists. The sample size was not calculated, as the aim was to include as many respondents as possible.

The structured questionnaire consisted of 25 questions in German (Table 1, Table 2, Table 3 and Table 4, questions translated into English).

The survey was administered as an eight-page A5 questionnaire brochure, an explanatory cover letter detailing the study’s purpose and significance, and a self-addressed prepaid return envelope. Participation in the survey was voluntary, and no identifiers and written informed consent were collected from respondents to ensure anonymity. Ethics committee approval was not required for this study, as the survey was directed to urologists, and additionally, no patient data were gathered.

The responses collected were entered into a Microsoft Excel spreadsheet (Microsoft Corporation, Redmond, WA, USA). Descriptive statistics were performed using means and standard deviations for continuous variables and frequencies and proportions for categorical variables. To test whether there were differences in the total number of first-time prescriptions of oral and IV BPs and denosumab per year according to age, gender, and years of clinical work experience, separate multinomial logistic regressions were performed. Pearson correlation coefficients were calculated to analyze the relationship between the percentage of correct answers and the age of the respondents, the number of MRONJ patients encountered, the number of first-time prescriptions of oral and IV BPs per year, and the interest in further education. Student’s *t*-tests and ANOVAs were conducted to test differences in the percentage of correct answers between genders, countries of practice, and years of work experience. All statistical tests were performed using the software “R” (The R Foundation, Vienna, Austria). *p* values of <0.05 were considered statistically significant.

## 3. Results

Among 187 returned questionnaires, 11 were incomplete and therefore excluded from the data analysis, yielding an overall response rate of 11.7%. Questions and the corresponding answers to baseline characteristics are provided in Table 1. The age of the participants ranged from 32 to 79 years, with a median age of 49 years (Table 1). Most respondents were male (83%), mainly practicing surgeons (66.5%). A total of 105 (59.7%) participants had been in practice for more than 16 years, reflecting the high prevalence of experienced urologists in the survey population. The primary work setting varied, with a spread of urologists working in solo practices, group practices, private clinics, and within the public sector at university or municipal, cantonal, and district hospitals.

The questions and responses about prescription frequencies of antiresorptive agents, the number of MRONJ patients encountered, and the implementation of dental referrals before starting antiresorptive therapy (AR) are presented in Table 2. Of the respondents, 77.3% (*n* = 136) and 58.5% (*n* = 103) stated that they never prescribe oral and IV BPs, respectively (Table 2). A total of 48.3% (*n* = 85) answered that they do not prescribe BPs. In contrast, 44.9% (*n* = 79) of all participants reported writing more than five first-time prescriptions of denosumab per year. Of 176 participants, 35.2% (*n* = 62) and 44.3% (*n* = 78) encountered about 1–5 cases of MRONJ in recent years related to BPs and denosumab, respectively. The vast majority (89.8%) reported implementing referrals to dentists for preventive dental screening before starting ARs, out of which 49 participating urologists additionally examine the oral cavity of the patients by themselves. Approximately 91.5% (*n* = 161) inform patients about the risk of developing MRONJ after exposure to BPs and denosumab. No statistically significant relationships were found between the total number of first-time prescriptions of antiresorptives (oral BPs, IV BPs, and denosumab) and age, gender, and years of clinical work experience.

The questions and answers of the questionnaire regarding self-assessment of knowledge about MRONJ, interest in continuing education in the field of MRONJ prevention, and knowledge about MRONJ are shown in Table 3. While 8.5% (*n* = 15) opined to have an excellent knowledge of MRONJ, the majority (42.6%) answered to be sufficiently informed. The question of whether MRONJ is recognized as an entity was affirmatively answered by 79.5% (*n* = 140) of the urologists. Most respondents identified exposed bone, swelling of the mandible, and tooth loosening as symptoms of MRONJ; however, fistula was singled out only by 54% (*n* = 95) of the participants. As shown in Table 3, 96% (*n* = 169) recognized that MRONJ does not only affect cancer patients. Interestingly, most respondents agreed that an ill-fitting prosthesis can trigger the development of MRONJ (81.8%) and that MRONJ lesions are clinically not well distinguished from malignant lesions (92.6%). A total of 87.5% of participants recognized that there is no defined period in which MRONJ occurs. Of the respondents, only 8.5% (*n* = 15) identified hypertension as a risk factor for MRONJ. Only a small subset of participants (13.1%) provided a misconceived answer to the question of whether antibiotic treatment often leads to the complete resolution of MRONJ.

The mean of the percentages of correct answers of each respondent was 70.9% with an SD of ±11.2%. There was a statistically significant positive correlation between the age of the respondents and the percentage of correct answers (*p* = 0.004). Moreover, a statistically significant positive correlation was observed between the number of encountered MRONJ patients and the rate of correct answers (*p* = 0.0013). Nevertheless, the number of first-time prescriptions of antiresorptives per year (*p* = 0.339) and the interest in further education in MRONJ prevention (*p* = 0.138) had no statistically significant influence on the percentage of correct answers. No statistically significant differences between the knowledge levels of Swiss, German, and Austrian urologists were recorded. Furthermore, gender and years of work experience did not have a statistically significant influence on the percentage of correct answers.

The answers to the question regarding the management of suspected MRONJ are depicted in Table 4. All respondents would refer patients with suspected MRONJ to an oral and maxillofacial surgeon, out of which 10.2% (*n* = 18) and 4.5% (*n* = 8) would also start with oral and IV antibiotics, respectively. A total of 16.5% (*n* = 29) would additionally refer for radiological examinations.

## 4. Discussion

The aging population and its impact on healthcare will undoubtedly pose significant challenges. Projections of a dramatic increase in cancer burden worldwide are foreseeable [34]. This will likely lead to an increased usage of antiresorptive agents and, consequently, an increase in MRONJ cases. In addition, emerging evidence suggests that a growing number of nonantiresorptive drugs might be involved in the development of MRONJ [35]. Therefore, it is of great importance to diminish the risk of MRONJ through preventive measures requiring a good level of knowledge among all health professionals involved in the care of patients at risk. A recently published practical guide for healthcare professionals summarized guidelines for the prevention and management of MRONJ [36].

The present study aimed to evaluate the knowledge level of Swiss, German, and Austrian urologists about MRONJ and whether they refer patients to dentists before starting AR for preventive dental measures.

In this survey, 96.6% of the participants were experienced urologists with more than 6 years of work experience. The percentage of correct answers correlated positively with the age of the respondents and the number of encountered MRONJ patients, respectively, as senior urologists may have witnessed more MRONJ cases and therefore acquired more knowledge about the condition compared with younger colleagues. Consequently, it cannot be excluded that younger participants and respondents with a notable lack of knowledge were more likely to miss the diagnosis of MRONJ and, therefore, gave a biased response regarding the number of MRONJ patients encountered. In contrast, a recent survey on MRONJ knowledge and management of MRONJ among germanophone dentists observed a statistically significant negative correlation between the age of the participants and the knowledge level [37].

The mean of the percentages of correct answers of each respondent was 70.9% with an SD of ±11.2%. Notably, only a small subset (8.5%) of the respondents singled out hypertension as a risk factor for MRONJ. This observation may be because hypertension has only recently been described as a risk factor for MRONJ [24]. Nevertheless, we considered the knowledge level sufficient, contrasting with the results of other studies among physicians in other countries. A study conducted in Brazil among physicians, nurses, and dentists showed a notable lack of awareness about MRONJ among the respondents [38].

Similarly, a survey of Japanese physicians showed a low knowledge level among general practitioners, internists, and orthopedists. Furthermore, less than 30% of the patients starting with AR were referred to dentists [39]. Similar findings of a survey conducted in Saudi Arabia demonstrated that more than half of the physicians never implemented preventive dental screenings [40]. In contrast, our study showed that about 89.8% of the respondents referred patients for preventive dental examinations. However, the abovementioned studies are only partially comparable with the present study since the questionnaires used were different and not standardized.

Preventive measures also include patient education about the risk factors of MRONJ before initiating AR to enable patients to maintain optimal oral health. In this survey, 91.5% (*n* = 161) of the participants informed the patients about the risk of MRONJ. In contrast, a study conducted in England, in which 23 MRONJ patients were interviewed, concluded that they were poorly informed about the risk factors of MRONJ and preventive strategies [41].

The present study has several limitations. Due to the relatively small sample size and low response rate of 11.7%, the findings may not represent all urologists in Switzerland, Germany, and Austria. Moreover, future surveys on the knowledge of MRONJ among healthcare professionals could differentiate between treatment purposes, schedules, and administration of low and high doses of oral and IV BPs and denosumab. Besides the well-known methodological disadvantages of survey research, such as recall bias, the possibility of informal discussions between the participants should be considered, as some questionnaires were sent to practitioners working in the same practice (e.g., group practice) or at the same hospital (e.g., university hospital).

## 5. Conclusions

In conclusion, this questionnaire-based survey showed that the participating urologists were sufficiently informed about MRONJ and, encouragingly, the vast majority referred patients to dentists for preventive dental examinations before starting AR. Nevertheless, we emphasize the importance of continuing education in MRONJ prevention for all healthcare professionals to further increase awareness of this condition and facilitate a multiprofessional approach and cooperation, thereby potentially improving patient safety and reducing the risk of developing MRONJ.

## Figures and Tables

**Table 1:** Questions and corresponding answers regarding baseline characteristics.

Questions		Country			
		Switzerland	Germany	Austria	Total
Country of practice, *n* (%)		60 (34.1)	76 (43.2)	40 (22.7)	176 (100)
Age (y), median (range)		45 (32–68)	54.5 (32–70)	50 (33–79)	49 (32–79)
Gender					
	Female, *n* (%)	11 (18.3)	13 (17.1)	6 (15)	30 (17)
	Male, *n* (%)	49 (81.7)	63 (82.9)	34 (85)	146 (83)
Years of clinical work experience (*y*)					
	1–5, *n* (%)	6 (10)	0 (0)	0 (0)	6 (3.4)
	6–15, *n* (%)	28 (46.7)	22 (28.9)	15 (37.5)	65 (36.9)
	16–25, *n* (%)	17 (28.3)	35 (46.1)	16 (40)	68 (38.6)
	>26, *n* (%)	9 (15)	19 (25)	9 (22.5)	37 (21)
Primary work setting					
	Solo practice, *n* (%)	12 (20)	13 (17.1)	23 (57.5)	48 (27.3)
	Group practice (two physicians), *n* (%)	5 (8.3)	21 (27.6)	1 (2.5)	27 (15.3)
	Group practice (more than two physicians), *n* (%)	11 (18.3)	34 (44.7)	5 (12.5)	50 (28.4)
	Private clinic, *n* (%)	0 (0)	0 (0)	1 (2.5)	1 (0.6)
	University hospital, *n* (%)	12 (20)	3 (3.9)	3 (7.5)	18 (10.2)
	Hospital ^1^, *n* (%)	19 (31.7)	5 (6.6)	7 (17.5)	31 (17.6)
	Rehabilitation center, *n* (%)	1 (1.7)	0 (0)	0 (0)	1 (0.6)
Clinical practice					
	Surgical urology >50%, *n* (%)	29 (48.3)	65 (85.5)	23 (57.5)	117 (66.5)
	Non-surgical urology >50%, *n* (%)	13 (21.7)	8 (10.5)	9 (22.5)	30 (17)
	Surgical and non-surgical urology equally, *n* (%)	18 (30)	3 (3.9)	8 (20)	29 (16.5)
Country of residency training					
	Switzerland, *n* (%)	49 (81.7)	0 (0)	0 (0)	49 (27.8)
	Germany, *n* (%)	10 (16.7)	75 (98.7)	3 (7.5)	88 (50)
	Austria, *n* (%)	1 (1.7)	1 (1.3)	36 (90)	38 (21.6)
	Other, *n* (%)	0 (0)	0 (0)	1 (2.5)	1 (0.6)

^1^ Includes municipal, cantonal, and district hospitals.

**Table 2:** Questions and corresponding answers regarding prescription of antiresorptive drugs, encountered MRONJ patients, and oral examinations before starting antiresorptive therapy (AR).

Questions		Country			
		Switzerland	Germany	Austria	Total
How many first-time prescriptions of oral bisphosphonates (BPs) do you give per year?					
	0, *n* (%)	48 (80)	61 (80.3)	27 (67.5)	136 (77.3)
	1–5, *n* (%)	10 (16.7)	8 (10.5)	12 (30)	30 (17)
	>5, *n* (%)	2 (3.3)	7 (9.2)	1 (2.5)	10 (5.7)
How many first-time prescriptions of intravenous (IV) BPs do you give per year?					
	0, *n* (%)	47 (78.3)	30 (39.5)	26 (65)	103 (58.5)
	1–5, *n* (%)	8 (13.3)	22 (28.9)	10 (25)	40 (22.7)
	>5, *n* (%)	5 (8.3)	24 (31.6)	4 (10)	33 (18.8)
How many first-time prescriptions of denosumab do you give per year?					
	0, *n* (%)	7 (11.7)	8 (10.5)	1 (2.5)	16 (9.1)
	1–5, *n* (%)	33 (55)	28 (36.8)	20 (50)	81 (46)
	>5, *n* (%)	20 (33.3)	40 (52.6)	19 (47.5)	79 (44.9)
How many MRONJ patients have you encountered (BP-associated)?					
	0, *n* (%)	8 (13.3)	11 (14.5)	7 (17.5)	26 (14.8)
	1–5, *n* (%)	11 (18.3)	38 (50)	13 (32.5)	62 (35.2)
	>5, *n* (%)	1 (1.7)	2 (2.6)	0 (0)	3 (1.7)
	Participants who do not prescribe oral and IV BPs, *n* (%)	40 (66.7)	25 (32.9)	20 (50)	85 (48.3)
How many MRONJ patients have you encountered (denosumab-associated)?					
	0, *n* (%)	30 (50)	35 (46.1)	12 (30)	77 (43.8)
	1–5, *n* (%)	22 (36.7)	30 (39.5)	26 (65)	78 (44.3)
	>5, *n* (%)	1 (1.7)	3 (3.9)	1 (2.5)	5 (2.8)
	Participants who do not prescribe denosumab, *n* (%)	7 (11.7)	8 (10.5)	1 (2.5)	16 (9.1)
Do you examine the patient’s oral cavity yourself before starting antiresorptive therapy (AR)?					
	Yes, *n* (%)	15 (25)	18 (23.7)	16 (40)	49 (27.8)
	No, *n* (%)	40 (66.7)	54 (71.1)	23 (57.5)	117 (66.5)
	Participants who do not prescribe antiresorptives, *n* (%)	5 (8.3)	4 (5.3)	1 (2.5)	10 (5.7)
Do you implement dental referrals before starting AR?					
	Yes, *n* (%)	49 (81.7)	71 (93.4)	38 (95)	158 (89.8)
	No, *n* (%)	6 (10)	1 (1.3)	1 (2.5)	8 (4.5)
	Participants who do not prescribe antiresorptives, *n* (%)	5 (8.3)	4 (5.3)	1 (2.5)	10 (5.7)
Do you inform your patients about the risk of MRONJ before starting AR?					
	Yes, *n* (%)	52 (86.7)	70 (92.1)	39 (97.5)	161 (91.5)
	No, *n* (%)	3 (5)	2 (2.6)	0 (0)	5 (2.8)
	Participants who do not prescribe antiresorptives, *n* (%)	5 (8.3)	4 (5.3)	1 (2.5)	10 (5.7)

**Table 3:** Questions and corresponding answers regarding awareness and knowledge about MRONJ.

Questions		Country			
		Switzerland	Germany	Austria	Total
How would you self-assess your knowledge about MRONJ?					
	Excellent, *n* (%)	4 (6.7)	7 (9.2)	4 (10)	15 (8.5)
	Good, *n* (%)	14 (23.3)	36 (47.4)	15 (37.5)	65 (36.9)
	Sufficient, *n* (%)	30 (50)	27 (35.5)	18 (45)	75 (42.6)
	Poor, *n* (%)	12 (20)	6 (7.9)	3 (7.5)	21 (11.9)
	No knowledge, *n* (%)	0 (0)	0 (0)	0 (0)	0 (0)
How would you classify your interest in continuing education in the field of MRONJ prevention?					
	Great, *n* (%)	26 (43.3)	23 (30.3)	21 (52.5)	70 (39.8)
	Moderate, *n* (%)	31 (51.7)	47 (61.8)	17 (42.5)	95 (54)
	No interest, *n* (%)	3 (5)	6 (7.9)	2 (5)	11 (6.3)
MRONJ is recognized as an entity. *Please select the correct answer.*					
	Correct *, *n* (%)	45 (75)	58 (76.3)	37 (92.5)	140 (79.5)
	Wrong, *n* (%)	15 (25)	18 (23.7)	3 (7.5)	36 (20.5)
Which of the following are symptoms of MRONJ? *Please select the correct answer(s).*					
	Exposed bone *, *n* (%) *of correct answers*	52 (86.7)	68 (89.5)	33 (82.5)	153 (86.9)
	Sudden attacks of severe, shooting pain affecting the trigeminal nerve, *n* (%) *of correct answers*	42 (70)	56 (73.7)	29 (72.5)	127 (72.2)
	Swelling of the temporal region, *n* (%) *of correct answers*	49 (81.7)	60 (78.9)	32 (80)	141 (80.1)
	Swelling of the mandible *, *n* (%) *of correct answers*	38 (63.3)	43 (56.6)	27 (67.5)	108 (61.4)
	Tooth loosening *, *n* (%) *of correct answers*	47 (78.3)	56 (73.7)	33 (82.5)	136 (77.3)
	Fistula *, *n* (%) *of correct answers*	25 (41.7)	42 (55.3)	28 (70)	95 (54)
MRONJ affects only cancer patients. *Please select the correct answer.*					
	Correct, *n* (%)	1 (1.7)	5 (6.6)	1 (2.5)	7 (4)
	Wrong *, *n* (%)	59 (98.3)	71 (93.4)	39 (97.5)	169 (96)
An MRONJ lesion associated with BPs. *Please select the correct answer(s).*					
	… is clinically well distinguished from a malignant lesion, *n* (%) *of correct answers*	53 (88.3)	73 (96.1)	37 (92.5)	163 (92.6)
	… is more likely to occur after exposure to IV BPs *, *n* (%) *of correct answers*	45 (75)	30 (39.5)	22 (55)	97 (55.1)
	… may be triggered from an ill-fitting prosthesis *, *n* (%) *of correct answers*	46 (76.7)	60 (78.9)	38 (95)	144 (81.8)
	… may develop even months after tooth extraction *, *n* (%) *of correct answers*	32 (53.3)	55 (72.4)	27 (67.5)	114 (64.8)
MRONJ can occur only after a certain period after the first exposure to antiresorptives. *Please select the correct answer.*					
	Correct, *n* (%)	6 (10)	13 (17.1)	3 (7.5)	22 (12.5)
	Wrong *, *n* (%)	54 (90)	63 (82.9)	37 (92.5)	154 (87.5)
Which are known risk factors related to MRONJ? *Please select the correct answer(s).*					
	Malignant disease *, *n* (%) *of correct answers*	34 (56.7)	60 (78.9)	27 (67.5)	121 (68.8)
	Diabetes mellitus *, *n* (%) *of correct answers*	49 (81.7)	65 (85.5)	37 (92.5)	151 (85.8)
	Renal failure *, *n* (%) *of correct answers*	20 (33.3)	31 (40.8)	13 (32.5)	64 (36.4)
	Osteoporosis *, *n* (%) *of correct answers*	33 (55)	49 (64.5)	26 (65)	108 (61.4)
	Hypertension *, *n* (%) *of correct answers*	6 (10)	8 (10.5)	1 (2.5)	15 (8.5)
	Comedication with corticosteroids *, *n* (%) *of correct answers*	47 (78.3)	57 (75)	30 (75)	134 (76.1)
Antibiotic treatment often leads to complete resolution of MRONJ. *Please select the correct answer.*					
	Correct, *n* (%)	7 (11.7)	4 (5.3)	12 (30)	23 (13.1)
	Wrong *, *n* (%)	53 (88.3)	72 (94.7)	28 (70)	153 (86.9)

* Correct answer according to the authors.

**Table 4:** Question and corresponding answers regarding the management MRONJ.

Question		Country			
		Switzerland	Germany	Austria	Total
*Please select your answer(s).* If MRONJ is suspected…					
	… start promptly with oral antibiotics, *n* (%)	4 (6.7)	5 (6.6)	9 (22.5)	18 (10.2)
	… refer promptly to an oral and maxillofacial surgeon, *n* (%)	60 (100)	76 (100)	40 (100)	176 (100)
	… start promptly with IV antibiotics, *n* (%)	2 (3.3)	4 (5.3)	2 (5)	8 (4.5)
	… refer promptly for radiological diagnostics, *n* (%)	10 (16.7)	11 (14.5)	8 (20)	29 (16.5)

## Data Availability

Raw data are available upon reasonable request.
